# An Empirical Model for Predicting Biodegradation Profiles of Glycopolymers

**DOI:** 10.3390/polym13111819

**Published:** 2021-05-31

**Authors:** Toma-Leonida Dragomir, Ana-Maria Pană, Valentin Ordodi, Vasile Gherman, Gabriela-Alina Dumitrel, Sorin Nanu

**Affiliations:** 1 Faculty of Automation and Computing, Politehnica University Timişoara, 2 Vasile Parvan bvd., 300223 Timişoara, Romania; toma.dragomir@upt.ro (T.-L.D.); sorin.nanu@upt.ro (S.N.); 2 Faculty of Industrial Chemistry and Environmental Engineering, Politehnica University Timişoara, 6 Vasile Parvan bvd., 300223 Timişoara, Romania; anamaria.pana@upt.ro (A.-M.P.); valentin.ordodi@upt.ro (V.O.); 3 Faculty of Civil Engineering, Politehnica University Timişoara, 2 Traian Lalescu str., 300223 Timişoara, Romania; vasile.gherman@upt.ro

**Keywords:** glycopolymer biodegradation, empirical model, model accuracy, dynamic nonlinear system

## Abstract

Pollution caused by plastic materials has a great impact on the environment. The biodegradation process is a good treatment solution for common polymers and biodegradation susceptible ones. The present work introduces new insight into the biodegradation process from a mathematical point of view, as it envisions a new empirical model for this complex process. The model is an exponential function with two different time constants and a time delay, which follows the weight loss profile of the polymer during the biodegradation process. Moreover, this function can be generated as the output variable of a dynamic exogenous system described through state equations. The newly developed models displayed a good fit against the experimental data, as shown by statistical indicators. In addition, the new empirical model was compared to kinetics models available in the literature and the correlation coefficients were closest to 1 for the new empirical model in all discussed cases. The mathematical operations were performed in the MATLAB Simulink environment.

## 1. Introduction

The pollution caused by plastic material waste during the last several decades has become one of mankind’s biggest problems [1,2,3]. It is noteworthy to mention the floating plastic island in the Pacific [4,5], debris contaminating soil and drinking water [6,7], birds and animals suffering injuries after consuming plastic fragments [8,9], etc. Although the scientific community has concentrated on finding solutions to this problem [10], up to date, there is no general method for properly disposing of plastic materials after use [8,11]. In the last decade, there has been an increasing recycling trend [12], which has spread mainly in Europe due to the UE politics towards environment preservation [13]. Also, new materials with enhanced biodegradable features have emerged as replacements for the classic ones especially in the food industry [14,15,16], although it is not enough to diminish the impact that plastic exerts on our environment [17,18,19].

Our group has been involved for more than 15 years in the synthesis and characterization of new polymeric materials with improved biodegradability based on renewable feedstock, such as sugars [20,21,22,23,24,25,26,27]. In the past few years, we have focused on the biodegradability process in terms of modeling the process and being able to determine the most important parameters that could influence the degradation of plastic materials derived from sugars in the presence of microorganisms. The biodegradation of sugar-based polymers was studied in liquid media using pure microbiological cultures [26,28], as well as natural microbiological consortia occurring in rivers [29]; also, the process was allowed to develop in static conditions (without shaking but at thermoset temperature) and in dynamic conditions inside a bioreactor [30,31,32]. In light of the good results, our research group was keen to investigate the development of the process following its dynamic behavior by using mathematical models that could provide additional information for improving the process and expanding it to common plastic materials.

Mathematical models can be applied to a variety of chemical and biochemical processes, taking into account the static and dynamic behavior of the system based on their parameters [33,34,35,36]. Simplifying theories involving characteristic function are often used to describe such systems: i.e., the kinetics of the biodegradation process is modeled by following the weight loss profile of the sample in time [37]. Nonetheless, the disadvantage of such simplifying statements is that certain intimate characteristics of the phenomena can be overlooked [38].

A dynamic exogenous mathematical model would be able to generate the characteristic function for chemical processes as variations of an output variable in time [39]. The literature in the field of mathematical modeling of chemical/biochemical processes tries to provide characteristic functions to describe the systems’ kinetics, based on the experimental results [40,41,42]. Thus, Benzekry et al. [40] comparatively analyze eight kinetics models in order to explain the evolution of tumor growth; the considered models are: exponential, exponential–linear, power law, Gompertz, generalized logistic, von Bertalanffy, and dynamic carrying capacity. Moreover, Angelucci et al. [41] proposed an integrated, well-explained, but rather complicated model for a two-step ex-situ bioremediation process of contaminated soil, while Tashiro and Yoshimura [42] developed a model which indirectly incorporates the logistic model for the synthesis of inducible enzymes.

As most processes encountered in the chemical and biochemical world involve complex transformations, there is always a gap left to be filled with proper mathematical models, which accurately describe the system behavior and its alteration of time.

This work proposes a new empirical mathematical model that can be parameterized in order to describe the weight loss profile of the sugar polymer samples during biodegradation under diverse conditions. The estimation of the model’s parameters used to model the kinetics of the biodegradation process was also undertaken. For testing the model, certain sets of experimental data were considered, either acquired in our laboratory or provided by literature in the field of polymer biodegradation. Accuracy parameters were calculated for each tested model considered. In addition, a dynamic model was constructed based on linear and non-linear variations of the weight loss profile during the biodegradation process.

## 2. Materials and Methods

### 2.1. Chemicals

D-mannose-based glycopolymers were synthesized and isolated previously [23,24,26,28]. The biodegradation process of the glycopolymers was studied according to the protocol established beforehand. The biodegradation process is generally described by the weight loss function (1).

(1) $$w\;\left(t\right)=\frac{m\left(0\right)-m\left(t\right)}{m\left(0\right)}\times 100$$

where *w*(*t*) is the weight loss at a given time *t* ≥ 0, (%); *m*(0) is the initial weight of the polymeric sample, (mass unit depends on context, e.g., grams); *m*(*t*) is the weight of the polymeric sample at a given time *t*, (mass unit depends on context, e.g., grams). The function *w*(*t*), *t* ≥ 0 reflects the process kinetics.

### 2.2. Mathematical Models Applied for the Kinetic Biodegradation Process

Usually, for the modeling of the kinetic of the Biodegradation Process the following four mathematical models are used:

Exponential Growth Model:

(2) $$w\left(t\right)=W_{\infty }\left[1-\exp\left(-t/T\right)\right]$$

where *w*(*t*) is the weight loss at a given time *t*, (%); $W_{\infty }$ is the weight-loss potential, (%); *t* is the time, (time unit depends on context); *T* is the time constant, (time unit depends on context).

Modified Gompertz Model:

(3) $$w\left(t\right)=W_{\infty }\cdot \exp\{-\exp\left[e\cdot R_{m}/W_{\infty }\cdot \left(\lambda -t\right)+1\right]\}$$

where *w*(*t*) is the weight loss at a given time, (%); $W_{\infty }$ is the weight-loss potential, (%); *R_m_* is the maximum value of the weight loss rate, (%/time unit); *t* is the time, (time unit depends on context); *λ* is the time constant of the process, (time unit depends on context).

Logistic Growth Model:

(4) $$w\left(t\right)=W_{\infty }/\left[1+\exp\left(-k\cdot t\right)\right)]$$

where *w*(*t*) is the weight loss at a given time, (%); $W_{\infty }$ is the weight-loss potential, (%); *t* is the time, (time unit depends on context); *k* is the time rate constant of the process, (time unit depends on context).

Gordon-Govind-Green-Imam-Shogren Model [43]

(5) $$w\left(t\right)=w_{\infty 1}[\frac{1}{a_{11}+a_{21}e^{-a_{31}t}+a_{41}e^{-a_{51}t^{2}}+a_{61}e^{-a_{71}t^{3}}}-\frac{1}{a_{11}+a_{21}+a_{41}+a_{61}}]\;+w_{\infty 2}\left[\frac{1}{a_{12}+a_{22}e^{-a_{32}t}+a_{42}e^{-a_{52}t^{2}}+a_{62}e^{-a_{72}t^{3}}}-\frac{1}{a_{12}+a_{22}+a_{42}+a_{62}}\right]$$

where *w*(*t*) is the weight loss at a given time, (%); $w_{\infty 1},w_{\infty 2}$ are the weight loss potentials, (%); *a*_1*i*_, *a*_2*i*_, *a*_4*i*_, and *a*_6*i*_ are the non-dimensional coefficients, (where *i* = 1, 2); *a*_3*i*_, *a*_5*i*_, and *a*_7*i*_ are the rate coefficients with the dimensions (time^−1^), (time^−2^), respectively, (time^−3^); *t* is the time, (time unit depends on context).

### 2.3. Theory/Calculation 

**Figure 1:**
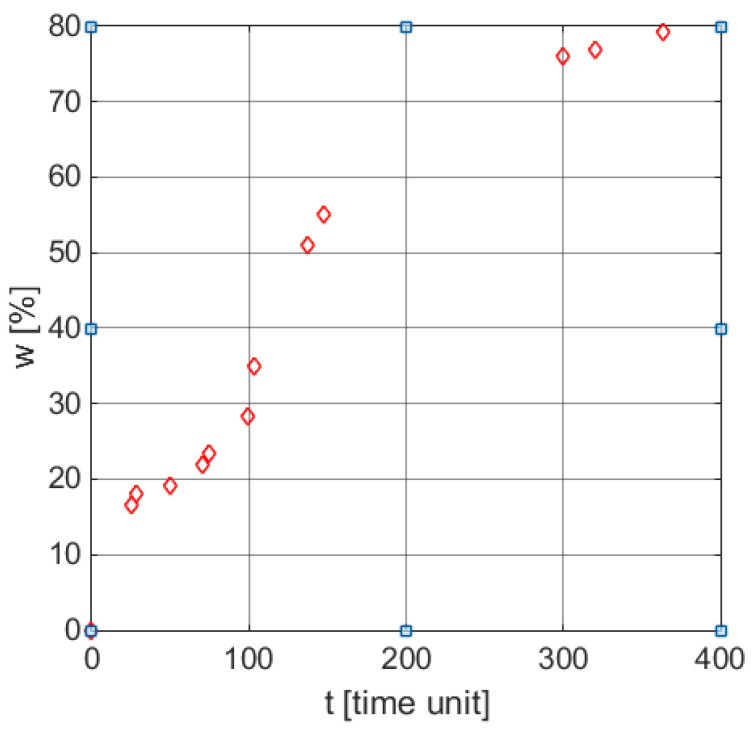
Weight loss profile in time for a typical biodegradation process [30].

The new empirical model for predicting biodegradation profiles of polymer samples proposed herein is a purely mathematic, black-box type model. It is formed by following the distribution of a weight loss profile for a biodegradation model (e.g., Figure 1) and assuming that it can be generally adequate to describe such processes. Two remarks can be emphasized:

- The variation of the weight loss values in time seems to correspond to a monotonous rising saturation tendency, as $w\left(t\right)=W_{\infty }$, where the weight loss potential $W_{\infty }$ has a finite value;
- The variation of the weight loss values in time reflects an additive property, which presents two exponential rising variations, separated in time by a time delay. This variation can be written similarly as the response to a step signal of a first-degree linear continuous system.

By combining these two assumptions, the following equation was obtained:

(6) $$w\left(t\right)=w_{1\infty }\left[1-\exp\left(-t/T_{1}\right)\right]+w_{2\infty }\;\left[1-\exp\left(-\left(t-\tau \right)/T_{2}\right)\right]\cdot \;\sigma \left(t-\tau \right)$$

where *σ*(*t*) is the unit step function; *w*(*t*) is the weight loss at a given time, (%); $w_{1\infty },w_{2\infty }$ are the weight loss potentials, (%); *T*_1_, *T*_2_ are the time constants, (days); *t* ≥ 0 is the time, (time unit depends on context); *τ* is the time delay (dead-time) of the process, (days).

Thus, the mathematical model (6) is a characteristic function of the process depending on five parameters aggregated in *P* vector (7): weight loss potentials $\left(w_{1\infty },w_{2\infty }\right)$ time constants (*T*_1_ and *T*_2_), and time delay (*τ*):

(7) $$P=[w_{1\infty },\;T_{1},\;\tau ,\;w_{2\infty },T_{2}]{}^{\prime }$$

The environment used for the modeling and simulation of the biodegradation process was the MATLAB R2018a Software Package. The model accuracy was estimated graphically and evaluated by calculating the following parameters: the relative absolute error (rAE), correlation coefficient (R), determination coefficient (R^2^), mean square error (SD), and the root mean square error (RMSE) [44].

## 3. Results and Discussions

### 3.1. Models of Characteristic Function Type for the Prediction of the Biodegradation Processes

From a mathematical perspective, the biodegradation process represents a distributed parameter process, which can be theoretically described by partial derivative equations. In practice, however, this approach was not yet exploited. The mathematical models used now to describe such processes are using characteristic functions, namely functions that describe the variation of a certain parameter over time (herein weight loss profile in time) and provide an insight into process kinetics. From a modeling perspective, this approach requires the choice of a characteristic function that may be parameterized and the fitting of its parameters according to the experimental data. Figure 1 illustrates the time profile weight loss corresponding to a biodegradation process, expressed by discrete points [43]. The goal of this work is to find the model for the weight loss parameter, i.e., a parametrized characteristic function.

All five models (2)–(6) are functions that may be parameterized. The adaptation of such a mathematical model to a certain biodegradation process consists of the evaluation of the models’ parameters based on the minimization of a criteria function attached to the model’s equation and the experimental data. Generally, combined regression and numerical minimization methods are applied to fit the parameters to minimize the criteria function, e.g., model (5) in [43], models (2), (3), and (4) in [30], model (4) in [29], and model (5) in [32].

**Figure 2:**
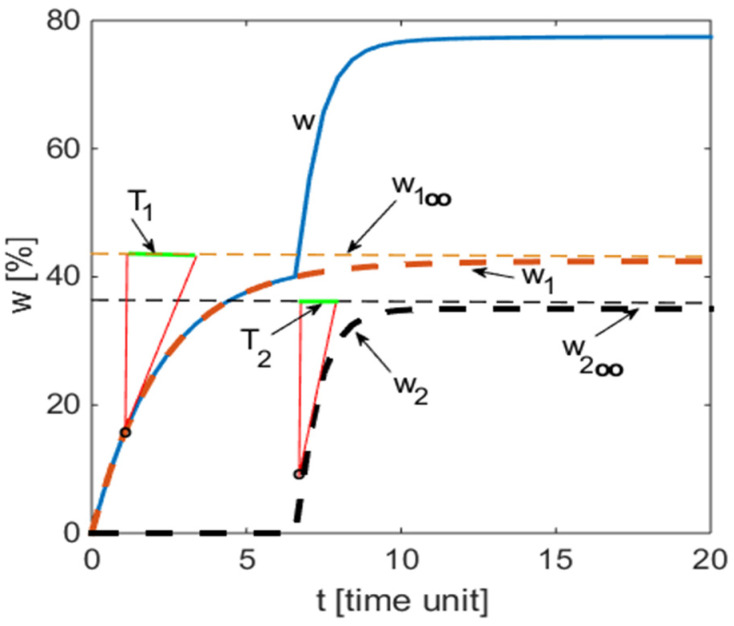
The time profile of weight loss depicted by Equation (6) and its components.

The new mathematical model (6) proposed herein envisions a general representation as the one presented in Figure 2. The time constants *T*_1_ and *T*_2_ appear in the graphical representation as subtangents cut on the asymptotes of the exponential growing curves.

From a systemic point of view, models (2)–(6) hide the fact that the biodegradation process corresponds to a dynamic system with distributed parameters. The idea of a “system with distributed parameters” is not addressed in this paper. We only limit to the issue of the behavior of a “dynamic system”. In this context, the starting point consists of two assumptions:

- The weight loss *w(t)* obtained experimentally in discrete time adequately describes the process and represents the output signal of the dynamic system.
- The biodegradation process is monitored from *t* = 0; the human operator is not involved in the process development after the process has started. Thus, the system evolves freely, independently from the operator’s will, from a certain initial state.

Based on the two working hypotheses stated above, the biodegradation process can be described using dynamic systems that generate signal *w*(*t*), as an exogenous signal corresponding to the experimental weight loss profile. This method is commonly adopted for control system synthesis, where the exogenous dynamic systems are used as reference signal generators or as disturbance signal generators [45,46]. The exogenous systems are obtained based on the shape of the signal they have to generate as free (natural) responses. Consequently, the exogenous systems are not physically real, but they generate signals issued by a physical system that is difficult to model. 

The signal represented by Equations (2)–(6) can be associated with different exogenous systems that can generate the weight loss profile *w(t)*: linear systems for signals (2) and (6), nonlinear systems for signals (3), (4), and (5). For instance, the dynamic nonlinear first-order model (8) can be associated to signal (4):

(8) $$W_{\infty }\cdot \dot{w}\left(t\right)+k\cdot W_{\infty }\cdot w\left(t\right)-k\cdot w^{2}\left(t\right)=0,w\left(0\right)=0$$

while a time delay system (9), with the state variable *x*_1_, …, *x*_4_, can be attributed to signal (6):

(9) $$\left\{ \begin{array}{c} \left\{ \begin{array}{l} {\dot{x}}_{1}(t)=0,\;x_{1}(0)=1\\ {\dot{x}}_{2}(t)=-T_{1}x_{2}(t),\;x_{2}(0)=1\\ {\dot{x}}_{3}(t)=0,\;x_{3}(0)=1\\ {\dot{x}}_{4}(t)=-T_{2}x_{4}(t),\;x_{4}(0)=1 \end{array}\right.\\ w(t)=w_{1\infty }(x_{1}(t)-x_{2}(t))+w_{2\infty }(x_{3}(t-\tau )-x_{4}(t-\tau ))(t-\tau ) \end{array}\right.$$

The parameters of models (8) and (9) are those of the characteristic functions (4) and (6).

It must be mentioned that although system (9) has four state equations, it is infinitely dimensional due to time delay (or dead-time) *τ*. Both models can be easily simulated by using the Simulink toolbox of MATLAB.

### 3.2. Validation of the Proposed Model

For all cases discussed herein, the nonlinear regression MATLAB function *nlinfit* (10) was used. This function requires as inputs, the *predictor,* namely the discrete time moments vector mt, the *regressor*, i.e., the corresponding weight loss values gathered in vector mw, the *estimator* represented by the model (6), and the initial values of parameters from (7) aggregated in the vector, vp. The output returned by *nlinfit* is the vector *P* of the estimated values from (7):

*P* = *nlinfit*(mt, mw, model(6), vp) (10)

In the sequel, the application of model (6) is limited to some experimental data corresponding to the biodegradation processes previously studied by authors [28,30] and to some data from other processes available in the literature linked to the development of a bacterial population [42,43].

#### 3.2.1. New Empirical Model Applied to Glycopolymer Biodegradation Processes

The newly developed model was applied to the biodegradation patterns of glycopolymers in different aqueous media. In Table 1, the first two rows present the experimental data obtained during the biodegradation process of a glycopolymer based on D-mannose and hydroxypropyl methacrylate inside a bioreactor fed with wastewater coming from the beer fabrication process [30]. According to Figure 2, the initial values of parameters of the newly proposed empirical model were established as vp = [40 2.2 5.2 38.1 1.15]’. By *nlinfit* regression function, the following values for the parameters were calculated:

*P* = [42.4331 2.2958 6.6296 35.0094 0.6614] (11)

Their values, substituted in (6), lead to the mathematical model (12):

(12) w(t)=42.4331[1−exp(−t/2.2958)]+35.0094[1−exp(−(t−6.6296)/0.6614]σ(t−6.6296)

The last two rows of Table 1 contain the calculated values of the weight loss using Equation (12) (CW), and the difference between the measured and the calculated values (ΔW), respectively. 

**Figure 3:**
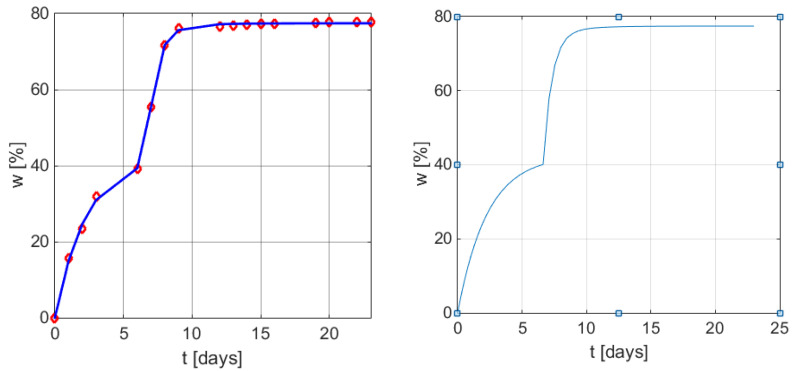
Graphical results of the weight loss profile obtained using the mathematical models (6) and (9). Left: measured values vs. values obtained from Equation (12) with MATLAB function *plot*. Right: *w*(*t*) curve using model (9) with parameters (11).

Figure 3 presents the graphical representation of the weight loss profile in time by applying the new empirical mathematical model (6), and (9), with the particularized parameters in (11). 

Table 2 presents the most important statistical indicators which show the accuracy of the used models (2), (3), (4), and (6), for the data depicted in Table 1. The first three rows are extracted from the literature [30]. 

The values of the statistical parameters from Table 2 can provide useful data concerning the adequacy of the used models. The values for model (6) particularized by (12), confirm that the new empirical model is the most suitable to characterize the biodegradation process.

This model seems to be very convenient for the modeling of the process because the two terms of Equation (12), in particular, and Equation (6), in general, could be associated with the components of the biodegradation process of these materials. It has been aforementioned in the literature [26,28,32] that these materials tend to first lose the sugar skeleton by biodegradation, a process illustrated by the first term of the mathematical model, and then the methacrylic network until saturation occurs, expressed by the second term of the proposed models. The time delay could thus be explained by the period of time required by the microorganism to become accustomed to using methacrylates as a carbon source for their metabolism.

The new empirical model was also applied for experimental data previously published in [32]. Table 3 presents the biodegradation weight loss profile for a D-mannose glycopolymer during the biodegradation process inside a bioreactor fed with water from the Bega River.

This time, model (6) takes the form of Equation (13).

(13) w(t)=35.448[1−exp(−t/0.7459)]+48.849[1−exp(−(t−12.3486)/14.6307)]σ(t−12.3486)

**Figure 4:**
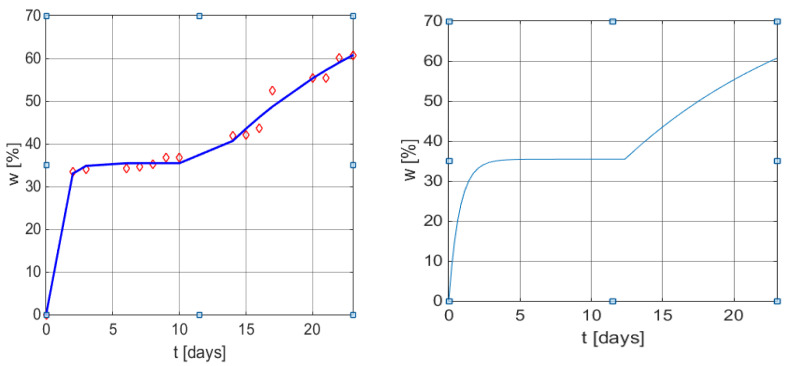
Graphical results of weight loss profile obtained by using the mathematical model (6) and (9). Left: measured values vs. values obtained from Equation (13) with function plot. Right: *w(t)* curve using model (9) with the same parameters as in (13).

The comparison with the original model (3) presented in the original article [32] is based on the correlation coefficients from Table 4 and the graphical illustrations from Figure 4.

#### 3.2.2. Validation of the New Empirical Model for on the Biodegradation Process of Polymers Derived from Natural Feedstock

Table 5 contains the data presented in the literature [43] corresponding to the biodegradation process of cornstarch and poly (β-hydroxybutyrate-co-β-hydroxyvalerate) in tropical water close to the shore. The first two rows refer to the coordinates of the seven points identified in Figure 1 from Gordon et al. [43], the values are presented in a white shade, and two other points obtained by visual interpolation, were required for the improvement of the regression calculus.

By applying the new empirical model to these data, Equation (14) is obtained.

(14) w(t)=22.6475[1−exp(−t/19.3845)]+57.7861[1−exp(−(t−91.7250)/68.5354)]σ(t−91.7250)

**Figure 5:**
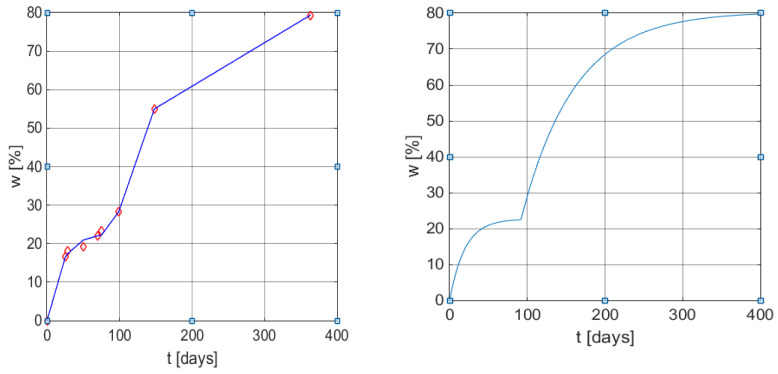
Graphical results of the weight loss profile obtained using the mathematical models (6) and (9). Left: measured values vs. values obtained from Equation (14) with MATLAB function *plot*. Right: *w*(*t*) curve using model (9) with the same parameters as in (14).

Table 6 presents the statistical indicators for this model. The graphical illustrations are shown in Figure 5.

#### 3.2.3. New Empirical Model Applied to Bacteria Population Growth

As the biodegradation process of the polymers can be linked to the development of the microbial substrate, we have attempted to use the new empirical model in order to explain the process of bacteria growth in a given environment. The time characteristic function for the process [42] is given by the colony-forming unit’s logarithm and corresponds to the general tendency profile presented in Figure 1. 

Table 7 presents the results obtained by applying the simulation of these processes based on Equation (6), while the customization is given by Equation (15).

(15) w(t)=2.8+1.2596[1−exp(−t/8.1116)]+4.6819[1−exp(−(t−7.4524)/6.7336)]σ(t−7.4524)

**Figure 6:**
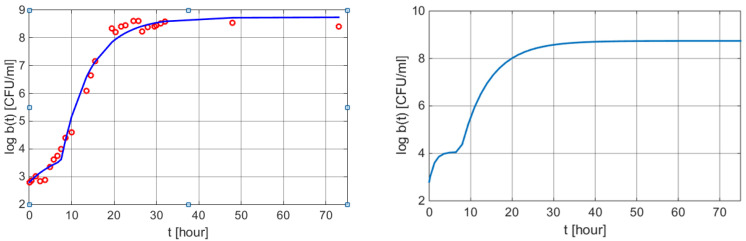
Graphical results of a weight loss profile obtained by using the mathematical models (6) and (9). Left: measured values vs. values obtained from Equation (15) with MATLAB function plot. Right: w(t) curve using the dynamic Simulink model (9) with the same parameters as in (15).

A supplementary term is added to the equation due to the fact that log b(0) = 2.8 CFU/mL. The corresponding graphical illustration is presented in Figure 6.

#### 3.2.4. Remarks on the Proposed Model

**Remark** **1.** *As presented in Section 3.2, when calling the MATLAB function nlinfit (8), we had to introduce the initial values of the five parameters referred to in Equation (7). Thus, in the case study from Section 3.2.1, the values vp = [40 2.2 5.2 38.1 1.15]’ were introduced, and nlinfit provided the values P = [42.4331 2.2958 6.6296 35.0094 0.6614]’. The initial values were estimated by drawing an approximate curve w(t) corresponding to measured weight loss and applying the features highlighted in Figure 2. The procedure was performed following the sequence:*

*(i)*  *The horizontal asymptote is drawn with an approximation for the first arc of the w(t) graph. The result is* $w_{1\infty }$ *.*
*(ii)*  *On the asymptote thus drawn, a subtangent is delimited by taking a point on the first arc. The length of the subtangent represents the value of T_1_.*
*(iii)*  *The angular point between the first and second arc is identified. The abscissa of this point represents the value of time delay* *τ.*
*(iv)*  *The horizontal asymptote is drawn for the second arc. Its value corresponds to* $w_{1\infty }+w_{2\infty }$ *. The value of* $w_{2\infty }$ *is obtained by subtracting from this sum the value obtained under point (i).*
*(v)*  *On the asymptote referred to in point (iv), a subtangent is delimited by considering a point on the second arc. Its length represents the value of T_2_.*

**Remark** **2.** *The values of the parameters in (6) provided by the nlinfit function are not unique. A renewed call of the function, with different or the same initial values, vp, nlinfit provides the vector P with values slightly different from the previous ones. This situation is due to the convergence of the algorithm used by nlinfit: iterative least-squares estimation. Consequently, the parameters’ values of models (9’)–(12) are not unique. The comparative statistical assessments made in each case as regards the resulting models were verified in many other recalculations.*

**Remark** **3.** *The results of model (6), as with models (2)–(5), become more acceptable when the number n of discrete values of w is determined experimentally, respectively the number of points (t, w(t)), is greater. If n is small, it is recommended to introduce additional points using an approximation curve similar to that in Remark 1. The case described in Section 3.2.2 is such an example.*

**Remark** **4.** *The parametrization of model (6) can be performed through nonlinear regression in different ways. For example, genetic algorithms or particle swarm optimization (PSO) method can be applied. In such cases, for estimating parameter fitness, functions can be used that penalize, depending on the acquisition moment, the errors given by the solution compared to measured values of w.*

**Remark** **5.** *The model (6) is always associated with a set of experimental data obtained from a biodegradation process, no matter if this is performed in a laboratory or in a natural environment. The parameters’ values for the model reflect the deployment conditions of the process. The applicability of the model (6) is not limited to biodegradable processes. It is applicable to other physical-chemical processes for which a characteristic variable evolves over time through points placed on curves such as the one in Figure 2.*

## 4. Conclusions

Mathematical models offer great insight into a process from both a practical and analytical point of view. The biodegradation process of a sugar-based polymer inside a bioreactor can be modeled with good accuracy using kinetic models, but from a dynamic point of view, the information they provide is scarce and/or unreliable. The new empirical model constructed herein by our group envisions the characterization of the process by means of weight loss *w*(*t*), as a time characteristic function. 

The proposed expression is a transcendental function with five parameters: two amplifications (gains), two time constants, and a time delay (dead-time). By proper parametrization of this model, the biodegradation profile of the studied samples in all discussed cases is well-reproduced. Moreover, the characteristic function can be associated with a dynamic model, i.e., an exogenous model used to generate it. The new model with two different time constants can explain the biodegradation pattern of the glycopolymers, as they tend to lose the sugar-based skeleton at first, by its assimilation due to microorganisms’ metabolism, and then the synthetic acrylate/methacrylate chain is altered by the microbiological environment. In mathematical terms, the first process, corresponding to the fast step of biodegradation can be attributed to the saccharidic chain degradation, while the longer stage belongs to the adjustment of the microorganism to consume the synthetic polymeric chain. Also, the new model was tested against the degradation of other polymeric materials as well as the bacteria population growth process and the results revealed that it presents a good fit with the experimental data.

For the assessment of the accuracy extent of the new model, the statistical parameters were calculated and compared to the kinetic models used in the literature; these results encourage us to believe that the new models used to describe the process are adequate and have the potential to be applied to other processes, which take into account the development of bacterial populations.

## Figures and Tables

**Table 1:** Results obtained by applying the new empirical model on the biodegradation data of the glycopolymer depicted in article [30].

**t (day)**	**0**	**1**	**2**	**3**	**6**	**7**	**8**	**9**	**12**
w(t) (%)	0	15.6630	23.3730	31.8670	39.1570	55.4520	71.5660	76.6870	79.6870
CW (%)	0	14.9836	24.6763	30.9465	39.3237	55.4341	71.7328	77.2042	77.2042
ΔW (%)	0	0.6794	1.3033	0.9205	−0.1667	0.0179	−0.1668	−0.5172	−0.5172
**t (day)**	**13**	**14**	**15**	**16**	**19**	**20**	**22**	**23**	
w(t) (%)	76.9280	77.1080	77.2290	77.4100	77.4700	77.7110	77.7110	77.8740	
CW (%)	77.2928	77.3466	77.3807	77.4026	77.4317	77.4355	77.4396	77.4406	
ΔW (%)	−0.3648	−0.2386	−0.1517	0.0074	0.0383	0.2755	0.2714	0.4334	

**Table 2:** Statistical indicators—comparative table for models (2), (3), (4), and (6) in the form of Equation (12).

Model	Statistical Parameters				
	Dispersion D	Root-Mean Square Deviation RMSD	Standard Deviation SD	Determination Coefficient R^2^	Correlation Coefficient R
(2)	0.456946	0.430067	0.67598	0.9164	0.9670
(3)	0.343164	0.322968	0.58579	0.9713	0.9753
(4)	0.26623	0.241527	0.50658	1.0040	0.9818
(6), respective (12)	0.2525	0.2376	0.5024	1.0010	0.9998

**Table 3:** Results obtained using the experimental data presented previously [32].

**t (day)**	**0**	**2**	**3**	**6**	**7**	**8**	**9**	**10**
w(t) (%)	0	33.339	34.055	34.1870	34.621	35.267	36.797	36.8
CW (%)	0	33.0208	34.8129	35.4366	35.4450	35.4472	35.4478	35.4479
ΔW (%)	0	0.3182	−0.7579	−1.2496	−0.8240	−0.1802	1.3492	1.3521
**t** **(day)**	**14**	**15**	**16**	**17**	**20**	**21**	**22**	**23**
w(t) (%)	41.924	42	43.581	52.419	55.4	55.4	60.17	60.647
CW (%)	40.6618	43.5446	46.2370	48.7515	55.3413	57.2543	59.0409	60.7095
ΔW (%)	1.2622	−1.5446	−2.6560	3.6675	0.0587	−1.8543	1.1291	−0.0625

**Table 4:** Statistical indicators—comparative data using models (4) and (6).

**Model**	**Statistical Parameters**				
	**Root-Mean Square Error RMSE**	**Mean Square Error** **SD**	**Determination Coefficient** **R^2^**	**Correlation Coefficient** **R**	**Relative Absolute Error** **rAE**
(4)	1.5534	2.4133	0.9987	0.9942	0.0015
(6), respective (12)	1.4959	2.2376	0.9892	0.9945	0.0188

**Table 5:** Results obtained using the weight loss profile data from [43]. The grey shaded values are the result of visual interpolation.

t (day)	0	25	28	50	70	75	99	148	363
mw (%)	0	16.5	18.0	19.1	22.0	23.33	28.33	55.0	79.33
CW (%)	0	16.4114	17.3055	20.9303	22.035	22.1747	28.33	55.0	79.33
ΔW (%)	0	0.0886	0.6945	−1.8303	−0.0355	1.1553	0.0	0.0	0.0

**Table 6:** Statistical indicators for the accuracy of model (6) in the case of experimental data from [43].

Model	Statistical Parameters				
	Root-Mean Square Error RMSE	Mean Square Error SD	Determination Coefficient R^2^	Correlation Coefficient R	Relative Absolute Error rAE
(6), respective (14)	1.7816	3.1739	0.9985	0.9975	0.0172

**Table 7:** Values of the statistical indicators obtained using the data from [42].

Process	Model	RMSE	MSE	R^2^	R	rAE
The temporal evolution of the logarithmic number of *Salmonella* spp. per unit mL in TSB at 30 °C, pH 5.3, and aw 0.997	(15), Figure 6	0.2640	0.0697	0.9753	0.9938	0.0245

## Data Availability

Not applicable.
